# Changes in Age at Diagnosis and Nutritional Course of Celiac Disease in the Last Two Decades

**DOI:** 10.3390/nu12010156

**Published:** 2020-01-06

**Authors:** Mónica Villanueva, Amaya Oyarzún, Bárbara Leyton, Mónica González, Elizabeth Navarro, Paulina Canales, Cristobal Ossa, María Paz Muñoz, Karla A. Bascuñán, Magdalena Araya

**Affiliations:** 1 Fellow, Pediatric Gastroenterology and Nutrition Program, Faculty of Medicine, University of Chile, Santiago, Chile, Clínica Alemana de Santiago, Chile; mvillanuevach@gmail.com; 2 Institute of Nutrition and Food Technology (INTA), University of Chile, Santiago, Chile; amaya.oyarzun@gmail.com (A.O.); bleyton@inta.uchile.cl (B.L.); karlabascunan@gmail.com (K.A.B.); 3 Hospital Roberto del Río, Santiago, Chile; monigonzaya@gmail.com; 4 Hospital Exequiel González Cortés, Santiago, Chile; elinavd@yahoo.es (E.N.); p.canalesramirez@gmail.com (P.C.); 5 Hospital Luis Calvo Mackenna, Santiago, Chile; jcossa@gmail.com (C.O.); pazmunioz@gmail.com (M.P.M.); 6 Department of Nutrition, Faculty of Medicine, University of Chile, Santiago, Chile

**Keywords:** celiac disease, age, nutritional status, follow up

## Abstract

The frequency of celiac disease (CD) has increased along time, with relevant changes reported in geographical variations, clinical presentation and nutritional repercussions. In recent years, some celiac patients are presenting overweight/obesity, but it is unclear how frequent this is and to what extent undernutrition remains a concern. This is relevant because CD tends to be overlooked in overweight patients. With this in mind, we assessed age at diagnosis, clinical characteristics and nutritional status of 155 celiac patients diagnosed between 1994–2017 in four pediatric hospitals in Santiago, Chile. Since 2003, the number of patients diagnosed has increased (*p* < 0.0033), coinciding with antitransglutaminase and antiendomysial antibodies becoming available to public health systems. In 2000, 4.5% of patients were asymptomatic at diagnosis, suggesting that active search is not routinely applied. Gastrointestinal symptoms plus failure to thrive were significantly more frequent under 2 years (*p* = 0.0001). Nutritional status has improved at diagnosis and during follow up, but undernutrition remains more frequent in children <2 and <5 years (*p* < 0.002 and *p* < 0.0036, respectively). Overweight at diagnosis was reported in 2002 and obesity in 2010. After initiating treatment, since 2010, patients changing from undernourishment to overweight has sometimes been observed after only 6 months on a gluten-free diet.

## 1. Introduction

The original descriptions characterized celiac disease (CD) as a condition that occurred especially during childhood, with severe diarrhea rapidly inducing malnutrition, but later, it was recognized as an autoimmune disorder, precipitated by exposure to dietary gluten (a protein present in wheat, rye, barley and oats) in genetically predisposed people [1,2]. The interaction between gluten, genetics, epigenetics mechanisms and microbiota are among the factors known to influence the appearance of the disease [1]. CD is known to have a broad clinical spectrum of presentations, large age range at which onset can occur and increased morbidity and mortality [2]. In the last few decades, there has been a steady global increase of obesity [3], and in recent years, celiac patients presenting with overweight/obesity have been described too, both at diagnosis and when put on a gluten-free diet (GFD) [4,5,6]. It is relevant to clarify to what extent undernutrition remains being a medical concern in CD, because the condition may be overlooked in overweight patients. As in many other countries, overweight/obesity has increased in Chile during the last decade [7], making our case interesting for assessing the relationship between CD and nutritional status. Thus, we set as objective to assess the clinical characteristics and nutritional status of celiac patients and their changes, if any, in the last ~two decades.

## 2. Materials and Methods 

We retrospectively assessed the clinical charts of patients with CD (CIE-10K90.0) diagnosed between 1994 and 2017, recording age at diagnosis, clinical presentation and nutritional status and their changes during treatment with GFD. Patients were diagnosed and followed in four main public pediatric hospitals in Santiago, Chile. CD has been assessed and described in patients of these hospitals in previous studies [8,9,10,11,12], providing insight about previous local conditions. Inclusion criteria were: (1) diagnosis based on at least one antibody measured [anti endomysium (EMA) and/or antitransglutaminase 2 (TTG) plus a duodenal histological lesion on endoscopic biopsies characterized by epithelial lymphocytosis and variable degrees of deepening of crypts and flattening of villi (Marsh 2 or more) [13]; (2) that weight and height data was available in at least two checkups within the first 12 months of GFD. Patients with additional diagnoses that could interfere with the diet or the nutritional status were excluded. Data at diagnosis was recorded in ad-hoc questionnaires, including family history, clinical characteristics, other diagnoses, serum immunoglobulin A (IgA), TTG, EMA, deamidated gliadin peptides (DGP) and histology on the duodenal biopsies. Adherence to GFD was assessed as reported by patients and their parents/caregivers during the routine, periodic controls. Medical records provided data on weight and height at each medical checkups. The Institutional Review Board of INTA, University of Chile, and that of each participating hospital approved the protocol.

Nutritional status was expressed as Z score of weight/age in patients less than one year of age, weight/height between 1–5 years and body-mass index (BMI) in those older than five years [14]. Data were analyzed by eANTHRO^®^ in children less than five years and ANTHROPLUS® in those older than five years [15,16]. Z scores up to −1SD defined nutritional risk, −1SD up to −2SD underweight and > −2SD malnutrition; −1SD to +1 SD normal nutritional status (eutrophics), +1SD up to +2SD overweight and >2SD obesity. Height/age <2SD defined short stature at all ages. No significant changes were detected in the number of cases excluded from analysis along the years; however, the number of patients diagnosed clearly varied along time. Therefore, analyses were performed on the total data and they were also separated into periods A (1994–2001, *n* = 15), B (2002–2009, *n* = 42) and C (2010–2017, *n* = 98).

Results were analyzed by time, age at diagnosis, clinical characteristics and nutritional status, taking data as a whole and also comparing periods A, B and C and their combinations. Analyses included descriptive statistics using STATA S/E v. 15.1 (Stata Corporation, College Station, TX), expressing results as frequency tables, mean, standard deviation and chi square. Chi square and Analysis of Variance (ANOVA, corrected by Bonferroni post-hoc) were used for categorical and continuous variables, respectively. Kruskal Wallis test was also used as needed. Significance was set at *p* < 0.05.

## 3. Results

Of the 201 charts identified, 155 met the inclusion criteria and entered analysis. Causes for exclusion were: incomplete data (*n* = 32), final diagnosis non-celiac gluten/wheat sensitivity (NCG/WS, 1), final diagnosis potential CD (*n* = 6), no biopsy (*n* = 4) and CD discarded (*n* = 3). Distribution of the charts not entering analysis did not show statistical differences between periods A, B and C. Of the 155 patients assessed, 15 (9.7%), 42 (27.1%) and 98 (63.2%) were diagnosed in each period, respectively; the number of diagnoses increased approximately three times between periods A and B and nearly six times between periods A and C.

### 3.1. At Diagnosis

Clinical characteristic at diagnosis are shown in Table 1. Of the 155 children assessed, 58.7% were younger than 5 years of age and 18.1% older than 10 years of age. Twelve patients (7.7%) had a first-degree relative diagnosed with CD. Only 7/155 (4.5%) were asymptomatic at the time of diagnosis, all of them were diagnosed after 2000 and assessed because they belonged to a risk group (mainly diabetes mellitus type 1 and Down syndrome).

Age at diagnosis significantly increased during the study period (ANOVA, *p* < 0.0033); this was apparent in 2003 and persisted until 2017. Among patients with gastrointestinal tract (GIT) presentations, mean age at diagnosis was 4.3 ± 3.6 years and most frequent symptom was diarrhea; among those with extra intestinal presentations, mean age was 6 ± 4.4 years and the most frequent presentation was failure to thrive (FTT). GIT symptoms were significantly more frequent in patients younger than 2 years of age (chi square, *p* = 0.0001), while FTT significantly diminished along the years (ANOVA, *p* = 0.003). TTG was measured in 2002; of the 78 patients who had it determined, 79.6% showed positive TTG while of 84 patients with EMA determinations, 89% were positive. As for histological assessment, 138 patients (89%) patients exhibited a mucosal lesion classified as Marsh ≥ 3 in the small intestinal biopsies, regardless the time elapsed between beginning of symptoms and diagnosis. Differences between GIT and extraintestinal presentations were not significant by histologic damage (93.8% and 94.1%, respectively).

**Figure 1:**
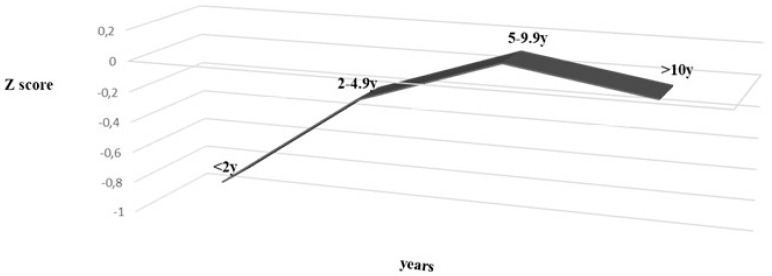
Nutritional status (Z score) and age at diagnosis, between 1999 and 2017 in 155 patients diagnosed celiac disease. (ANOVA *p* = 0.002).

Nutritional status at the time of diagnosis was related with age and year of diagnosis. There was a significant increase of age at diagnosis and improvement of nutritional status along the study period (ANOVA, *p* < 0.002) (Figure 1). Children under 2 and under 5 years of age had significantly lower Z score when compared with older children (*p* < 0.002 and *p* < 0.0036, respectively).

Among children that presented with GIT symptoms, clinical presentation was not associated with nutritional status (*p* = 0.87). Instead, undernutrition [14] was more frequent among those presenting with extra intestinal manifestations (*p* < 0.002).

### 3.2. Nutritional Changes during Follow Up on GFD

**Figure 2:**
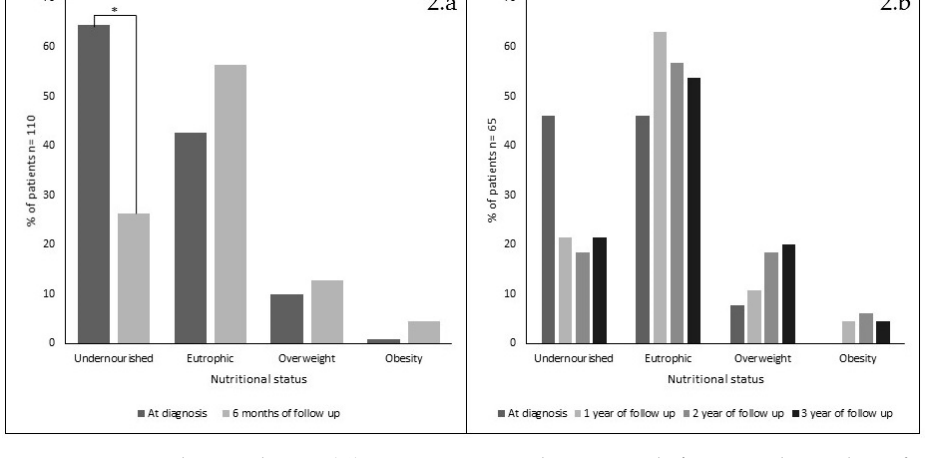
Nutritional status changes (%) in 110 patients, at diagnosis and after 6 months on gluten-free diet (1994–2017) (panel 2.a) and in 65 patients at diagnosis and after 1, 2 and 3 years on GFD (panel 2.b). * = chi square *p* < 0.001).

As a whole, there were no significant differences detected when each nutritional indicator was compared in periods A, B and C. Acute effects of treatment was analyzed in 110/155 patients with nutritional data available at diagnosis and after 6 months on GFD (Figure 2, panel 2.a); undernourishment significantly decreased (*p* = 0.001), while eutrophic and overweight children showed no significant increased trend (both *p* > 0.05). Overweight children were present only in periods B and C and obese patients only in period C, changing from 1 (at diagnosis) to 5 after six months on GFD.

Analysis of 65/155 patients who had nutritional data available at diagnosis and after 1, 2 and 3 years on GFD is shown in Figure 2, panel 2.b, no significant changes were detected. Only 12 patients had annual data available during the 18-year period analyzed. Their nutritional progression showed a steady improvement along the years, similar to the results obtained when data were analyzed yearly or by periods (data not shown). However, it is noticeable that only after 2010, some patients improved their Z score classification from undernourishment to overweight after only 6 months on GFD (not shown).

## 4. Discussion

### 4.1. Frequency of Diagnosis and Clinical Characteristics

Results show a significant increase of CD diagnosis after 2003 (ANOVA, *p* < 0.0033). Since the early descriptions of CD in the 1950s [17,18] diagnosis of this disease is reached by means of small intestinal biopsies in the hospitals participating in this study, making it unlikely that an initial lack of awareness on the part of medical professionals in charge of the patients was a relevant factor influencing the number of patients diagnosed. Additionally, comparison of the chart exclusion rate in the three study periods (A, B and C) showed no significant changes. This suggests that the ~six-fold increase of patients diagnosed may represent a true increase of diagnosis of the CD. The fact that such increase coincides with the introduction of TTG and EMA determinations to the national public health systems suggests that their availability was indeed a relevant factor to improve diagnosis. This effect of TTG/EMA utilization has also been described by other authors [2] and supports the current experts’ opinion that measuring TTG is the best option for screening CD [19]. Unfortunately, results of this study do not clarify the controversy about its utility during follow up [20]. The small number of asymptomatic patients (7/155, 4.5%) and of first-degree relatives detected (Table 1) suggests that active search is not a routine practice in the hospitals assessed. A clear limitation in this study is the incomplete data available in the charts along follow up. Nevertheless, when missing clinical and nutritional data were compared in periods A (1994–2001), B (2002–2009) and C (2010–2017), differences were not significant.

### 4.2. Clinical Characteristics

It is interesting that in patients under 18 years of age, diagnosis before 5 years of age remains in more than 50% of cases. Before 2002, mean age of diagnosis was at younger ages (mean 1.9 y), similar to figures previously reported in Chile [11,21] and younger than those reported in other countries in Latin America, where diagnosis is described at a mean age of ~6 y [22]. However, in 2002, the mean age of diagnosis increased (mean 5.1 y during 2003–2017), following the shift to older ages described in other studies [23].

GIT manifestations were more frequently the leading symptoms in children younger than 2 years of age. Abdominal pain was a main symptom in the 32 overweight patients detected, concurring with reports by Reilly [24] and Venkatasubramani [25]. Agreeing with previous local studies [10], a high percentage of patients (89%) exhibited intense histological damage in the small intestinal mucosa at diagnosis. Previous studies in Chile show that the frequency and distribution of Human Leucocyte Antigen (HLA) -DQ2 and -DQ8 differ from those reported in Europe [26], HLA-DQB1*0301 - HLA-DQA1*302 being more frequent, but we have no data suggesting that the clinical presentation of the disease may be influenced by these genetic differences.

### 4.3. Nutritional Status

At diagnosis and consistent with younger patients having more severe clinical presentations, nutritional status was significantly poorer in younger children (<2 and <5 years of age, chi square *p* < 0.002 and *p* < 0.0036, respectively). In a recent study, Mansueto et al. compared patients with CD and NCG/WS, reporting similar frequencies of undernutrition in younger children, in both conditions [27]. FTT, one of the most frequently reported extra intestinal manifestation [28], showed a decreasing trend at older ages (chi square, *p* > 0.05).

As for overweight/obesity, since description of CD in an obese patient [5], reports of obesity have increased in this condition [24,25,29,30]. In Chile, obesity at all ages has greatly increased in the last decades [7,31], currently being reported at 40% in preschoolers [6,32] and 75% in the adult population [31]. In this study though, overweight/obesity is less frequent than in the general pediatric population, which agrees with a recent report by Schilling et al [6]. We would like to speculate that periodic medical controls have a positive educational influence, teaching the patient how to keep a more nutritionally adequate GFD. It is most relevant that in 2010, after 6 months on GFD, some patients increased their Z score from undernourishment to overweight, emphasizing that adequate nutritional follow up must include not only caring for eliminating dietary gluten, but also for maintaining a healthy nutritious diet. This issue represents a huge challenge for the public health system, where the number of dietitians is often insufficient to provide regular care to all patients that require special diets for treatment.

## 5. Conclusions

Results of this study show that during the last two decades, the diagnosis of CD has increased six times and diagnosis tends to be at older ages, which agrees with recent reports that describe a steady increase in the number of diagnoses identified, even in geriatric patients [33,34]. In our study, the increase of diagnosis coincides with TTG and EMA becoming available to the public health systems. Nutritional status of celiac patients is better both at diagnosis and during follow up on GFD. GIT symptoms and undernutrition remain more frequent in younger children, especially under 2 and 5 years of age. Obesity is increasingly found at diagnosis, although its frequency is lower than in the general population. Since 2010, nutritional status changing from undernourishment to obesity is sometimes observed after only 6 month GFD.

## Figures and Tables

**Table 1:** Clinical characteristics in the 155 patients diagnosed with celiac disease between 1994 and 2017.

	Period A 1994–2001 (*n* = 15/155) mean ± SD	Period B 2002–2009 (*n* = 42/155) mean ± SD	Period C 2010–2017 (*n* = 98/155) mean ± SD	Total (*n* = 155) mean ± SD	*p* *
Age at diagnosis (y)	1.9 ±1.2	5.1 ± 3,8	5.7 ±4,3	5.1 ± 3.6	0.003
Sex. Girls, *n*(%)	10 (66.7%)	27 (64.3%)	63 (64.3%)	100 (64.5%)	0.983
Gastrointestinal symptoms, *n* (%)					0.090
Diarrhea	12(80)	17 (40.5)	57 (58.1)	86 (55.4%)	
Vomiting	4 (26.7)	10 (2.4)	18 (18.4)	32 (20.65%)	
Abdominal pain	1 (6.7)	5 (11.9)	28 (28.6)	34 (21.94%)	
Abdominal distension	7 (46.7)	9 (21.4)	33 (33.7)	49 (31.61%)	
Constipation	0 (0.0)	5 (11.9)	18 (18.4)	23 (14.84%)	
Total	13 (86.7)	26 (61.9)	75 (76.5)		
Extraintestinal symptoms, *n* (%)					
Failure to thrive	9 (60)	22 (52.3)	32 (32.7)	63(40.65%)	0.020
Short stature	2 (13.3)	8 (19.1)	22 (22.5)	32(20.65%)	
Anemia	3 (20)	8 (19.1)	8 (8.2)	19(12.26%)	
Wasted bottoms	0 (0.0)	4 (9.5)	9 (9.1)	13(8.39%)	
Irritability/Apathy	2 (13.3)	0 (0.0)	4 (4.1)	6(3.87%)	
Dermatitis Herpetiformis	0 (0.0)	0 (0.0)	3 (3.1)	3(1.94%)	0.003
Oral aphthae	0 (0.0)	0(0.0)	2 (2.0)	2(1.29%)	
Enamel hypoplasia	0 (0.0)	1 (2.4)	1 (1.0)	2(1.29%)	
Pubertal delay	0(0.0)	0 (0.0)	1 (1.0)	1(0.65%)	
Weakness	0 (0.0)	1 (2.4)	0 (0.0)	1(0.65%)	
Total	13 (86.7)	32 (76.2)	58 (59.2)		
Asymptomatic	0 (0.0)	2 (4.8)	5 (5.1)	7(4.52%)	
Presenting comorbidities	1 (6.7)	8 (19.1)	24 (24.5)	33(21.2%)	
Positive family history	0 (0.0)	8 (19.1)	4 (4.1)	12(7.74%)	
Belongs to risk group	1 (6.7)	7 (16.7)	18 (18.4)	26(16.7%)	

* *p* = (chi square).
